# Supplementary material for: PTPN22 R620W gene editing in T cells enhances low-avidity TCR responses
Source: eLife. 2023 Mar 24;12:e81577. doi: 10.7554/eLife.81577 (PMC10065793; doi:10.7554/eLife.81577)
Supplement: Figure 1—source data 1. [file elife-81577-fig1-data1.zip › Anderson et al Western Blot images labeled.pptx]

## Slide 1
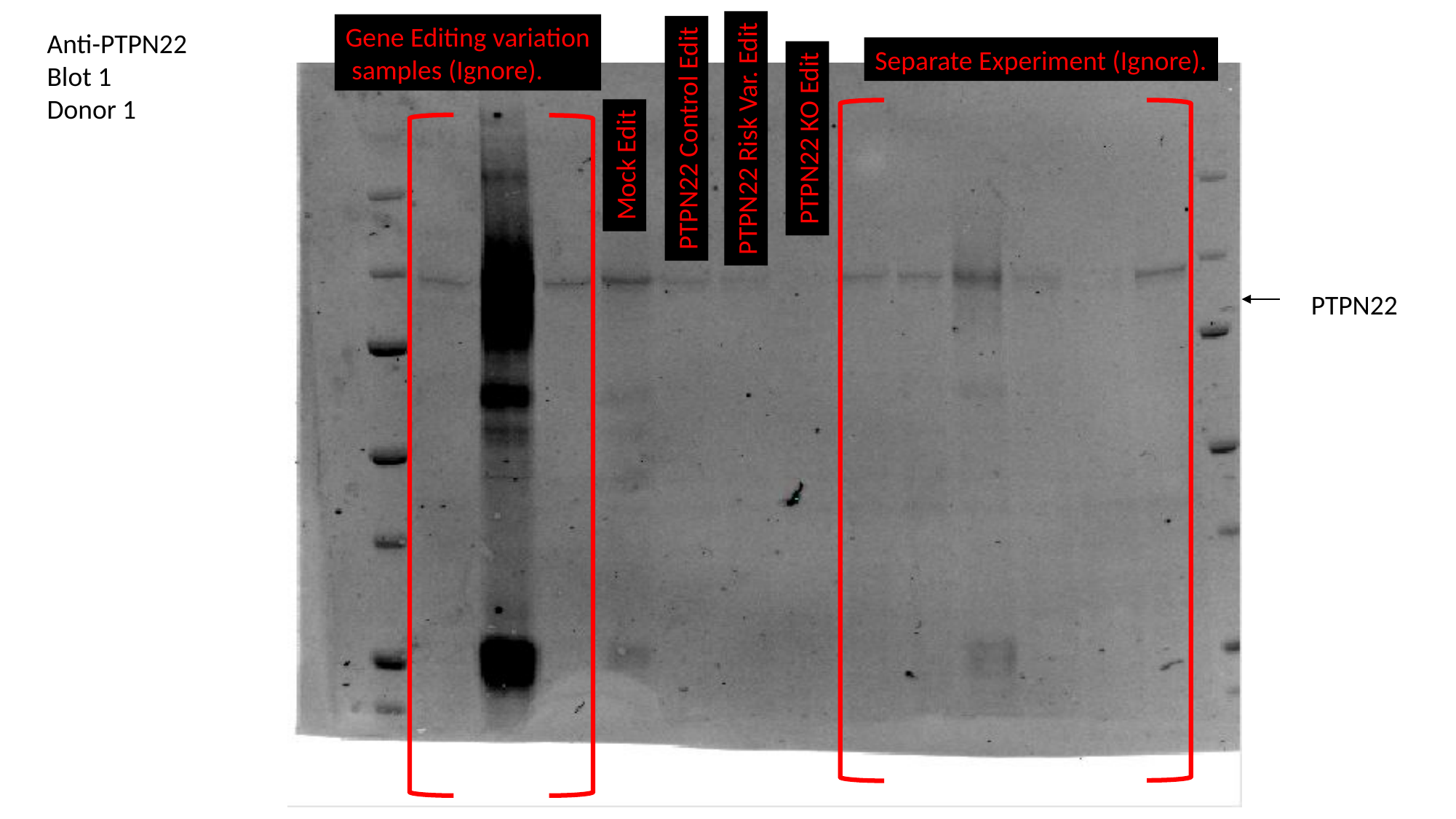

Gene Editing variation
 samples (Ignore).
Anti-PTPN22
Blot 1
Donor 1
Separate Experiment (Ignore).
PTPN22 Control Edit
PTPN22 Risk Var. Edit
PTPN22 KO Edit
Mock Edit
PTPN22

## Slide 2
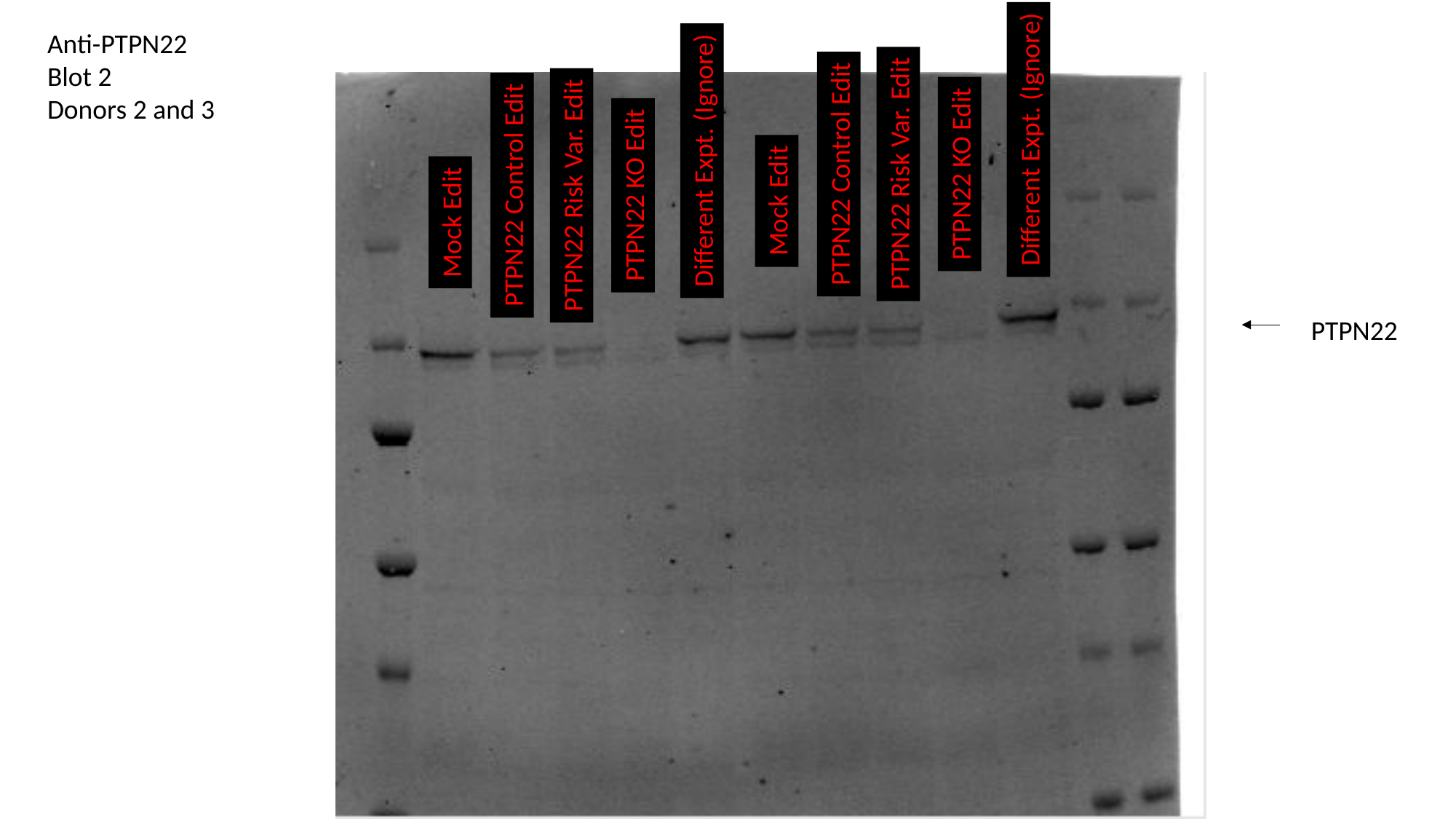

Anti-PTPN22
Blot 2
Donors 2 and 3
Different Expt. (Ignore)
Different Expt. (Ignore)
PTPN22 Control Edit
PTPN22 Risk Var. Edit
PTPN22 KO Edit
PTPN22 Control Edit
PTPN22 Risk Var. Edit
PTPN22 KO Edit
Mock Edit
Mock Edit
PTPN22

## Slide 3
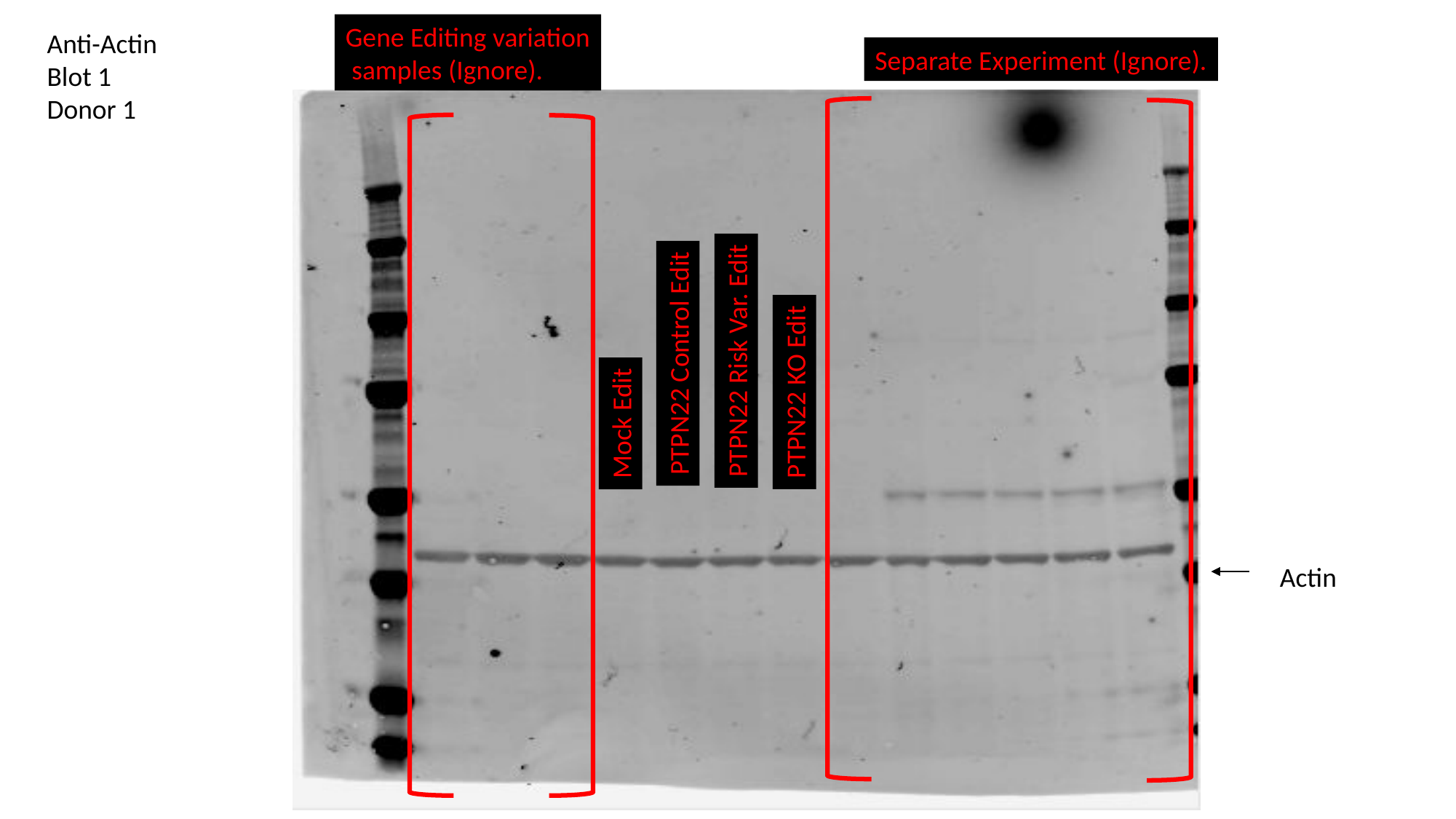

Gene Editing variation
 samples (Ignore).
Anti-Actin
Blot 1
Donor 1
Separate Experiment (Ignore).
PTPN22 Risk Var. Edit
PTPN22 Control Edit
PTPN22 KO Edit
Mock Edit
Actin

## Slide 4
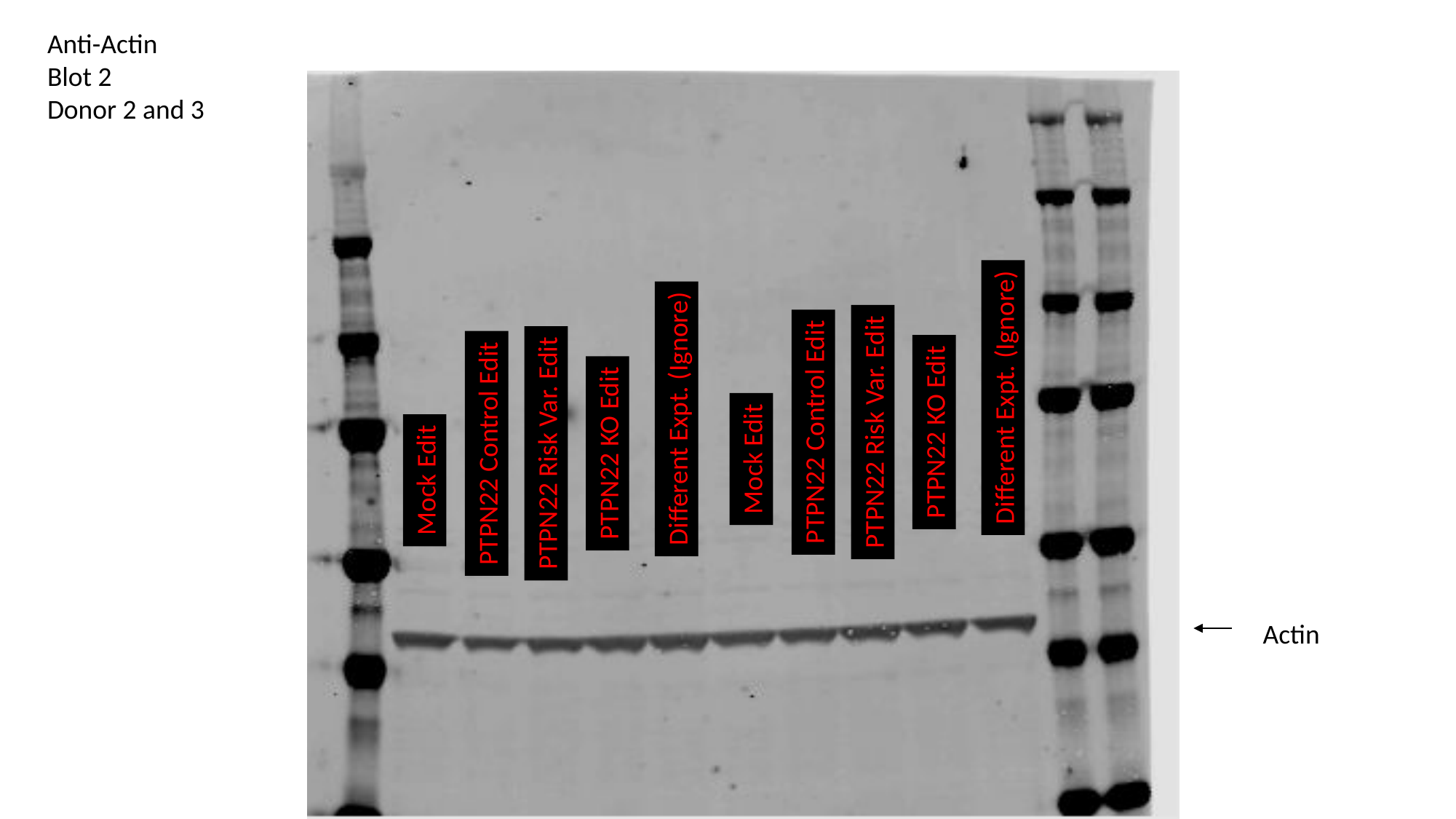

Anti-Actin
Blot 2
Donor 2 and 3
Different Expt. (Ignore)
Different Expt. (Ignore)
PTPN22 Control Edit
PTPN22 Risk Var. Edit
PTPN22 KO Edit
PTPN22 Control Edit
PTPN22 Risk Var. Edit
PTPN22 KO Edit
Mock Edit
Mock Edit
Actin
