# Supplementary material for: PTPN22 R620W gene editing in T cells enhances low-avidity TCR responses
Source: eLife. 2023 Mar 24;12:e81577. doi: 10.7554/eLife.81577 (PMC10065793; doi:10.7554/eLife.81577)

Anti-PTPN22
Blot 1
Donor 1

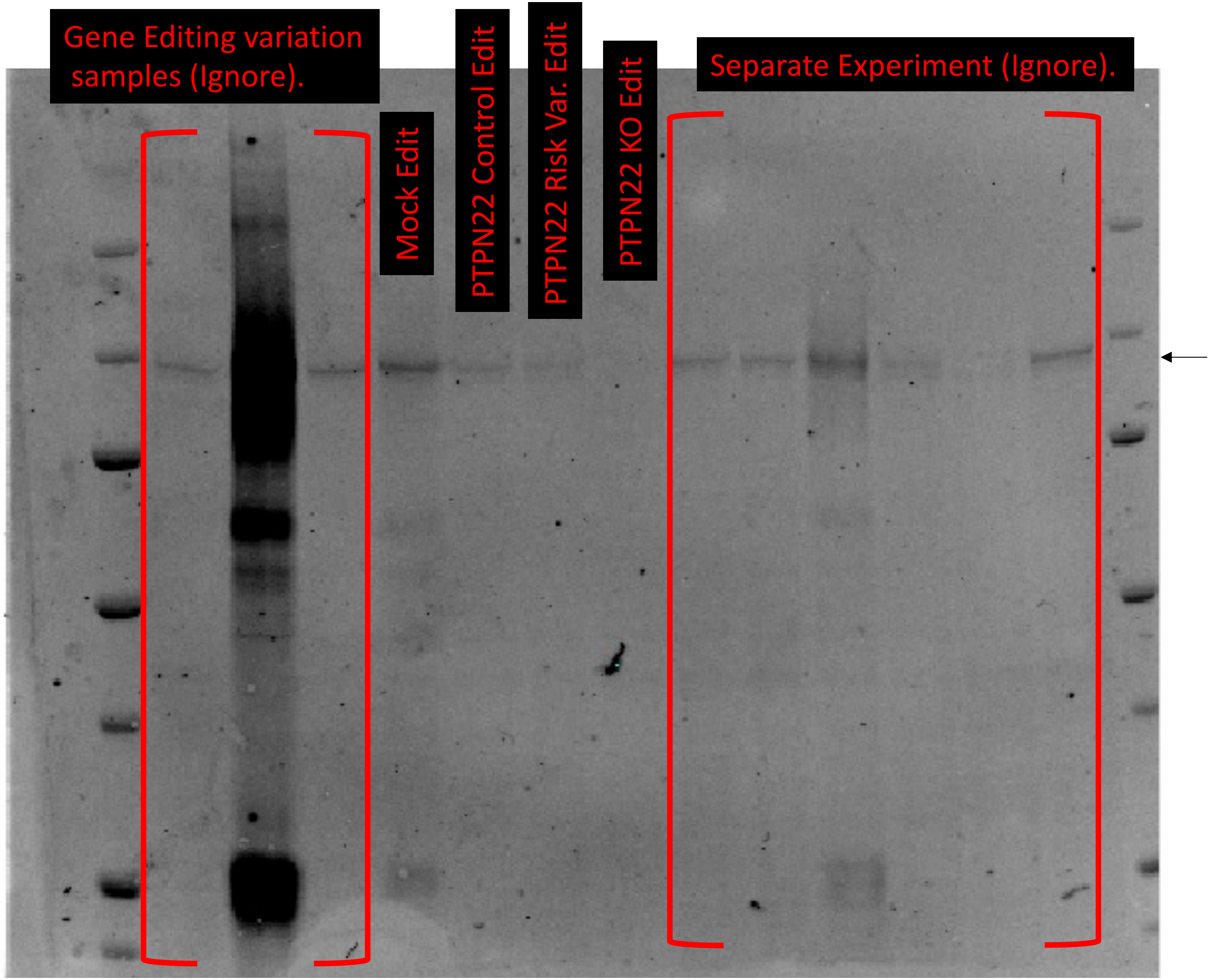

PTPN22

Anti-PTPN22
Blot 2
Donors 2 and 3

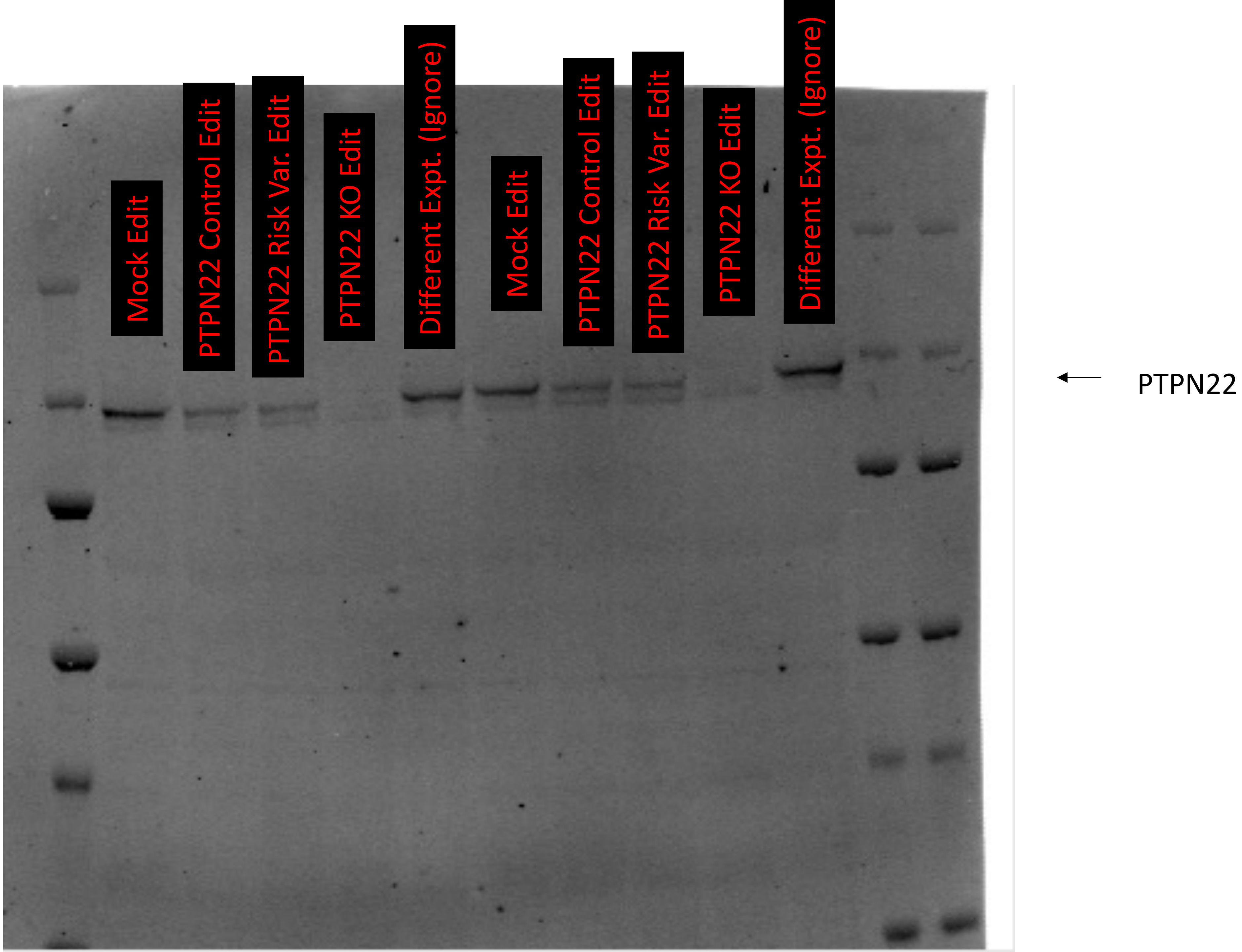

Anti-Actin
Blot 1
Donor 1

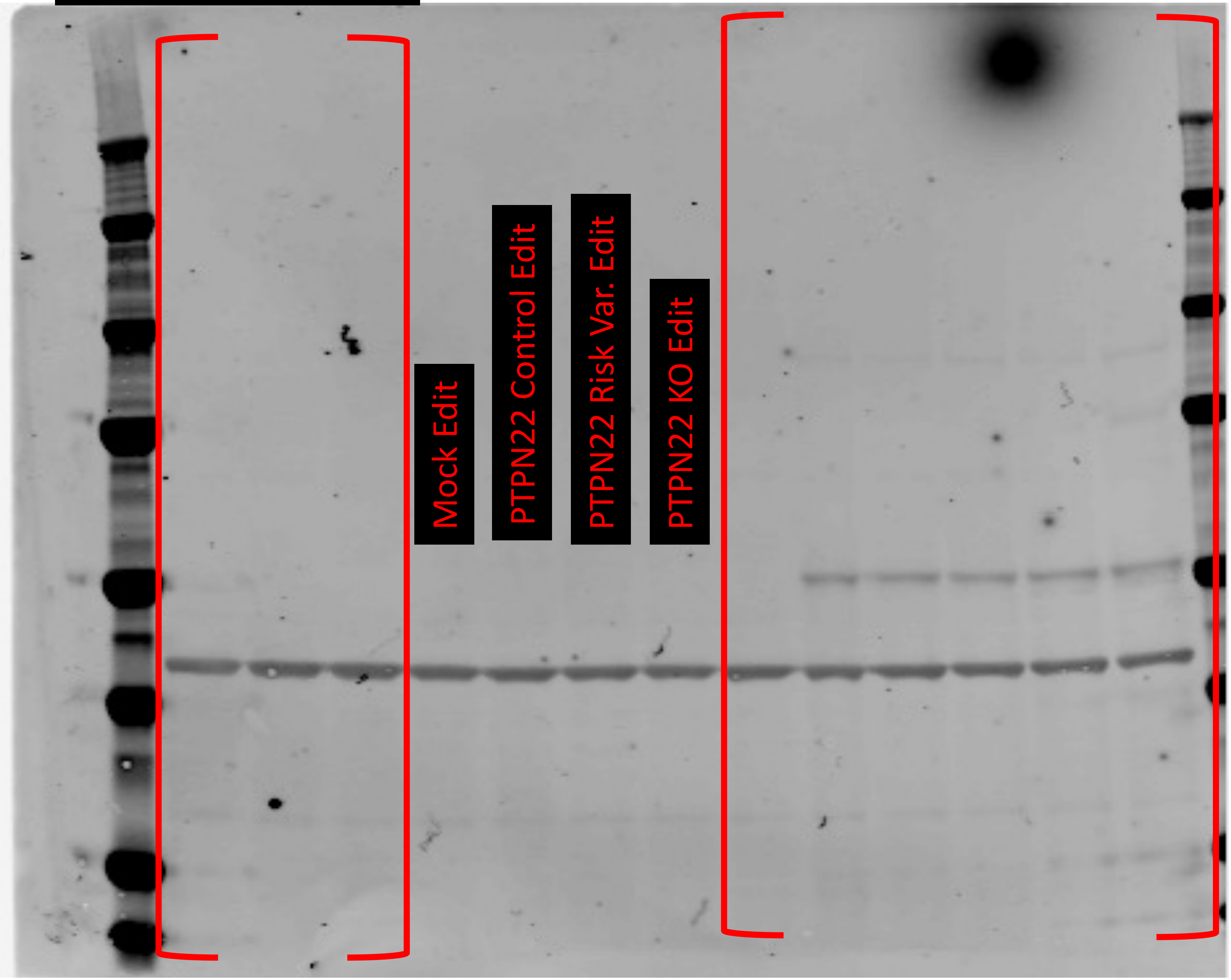

Anti-Actin
Blot 2
Donor 2 and 3

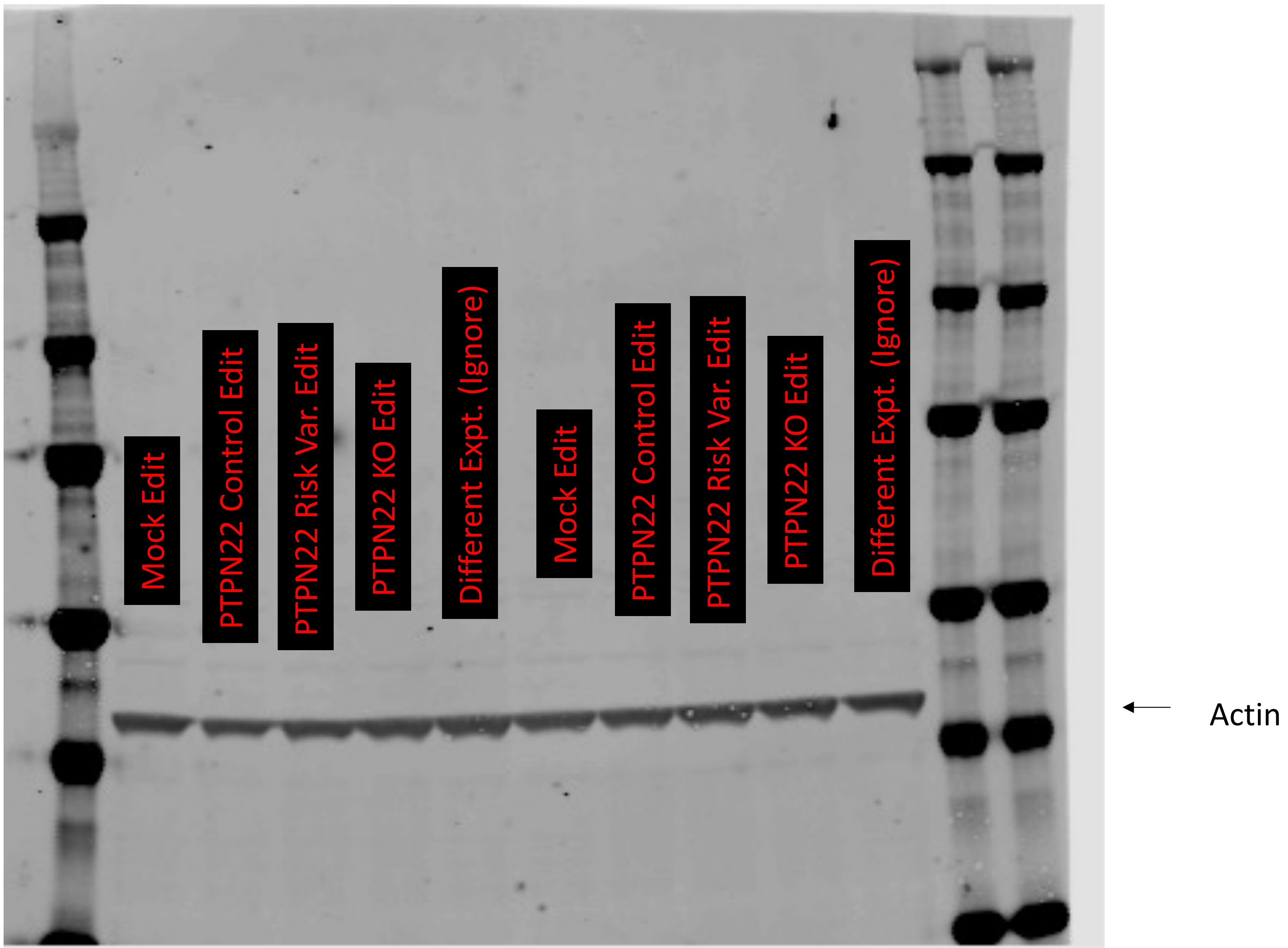

Supplement: Figure 1—source data 1. [file elife-81577-fig1-data1.zip › Western Blot Images Labeled.pdf]
